# Independently validating a novel modification to the Single Molecule Array technology in a pilot Alzheimer’s disease cohort

**DOI:** 10.1002/alz.095520

**Published:** 2025-01-09

**Authors:** Deborah O T Alawode, Syrena Fernandes, Amanda J Heslegrave, Cheuk W Kan, David Rissin, David C Duffy, Henrik Zetterberg

**Affiliations:** ^1^ UK Dementia Research Institute at UCL, London United Kingdom; ^2^ Quanterix, Lexington, MA USA; ^3^ UCL Queen Square Institute of Neurology, London United Kingdom; ^4^ Institute of Neuroscience and Physiology, Sahlgrenska Academy at the University of Gothenburg, Gothenburg Sweden; ^5^ Dementia Research Centre, Department of Neurodegenerative Disease, UCL Queen Square Institute of Neurology, University College London, London, United Kingdom, London United Kingdom; ^6^ UCL Institute of Neurology, Queen Square, London United Kingdom

## Abstract

**Background:**

A novel improvement to Quanterix Corporation Single molecule array (Simoa) SR‐X instrument has been shown to offer up to 100‐fold increased sensitivity compared with standard Simoa HD‐X and SR‐X instruments. We independently validated this increased sensitivity using the Simoa interleukin 17A (IL17A) assay.

**Method:**

IL17A was measured in 32 paired Alzheimer’s disease (AD; n = 13) and control (CTRL; n = 19) serum and cerebrospinal fluid (CSF) samples. Mean IL17A concentrations were compared in both biofluids measured using four Simoa instruments ‐ one HD‐X instrument and three SR‐X instruments modified to improve the efficiency of reading beads.

**Result:**

HD‐X quantified IL17A in 0% of CSF samples and 37.5% of serum samples, with mean serum IL17A concentrations of 0.126pg/mL in AD and 0.228pg/mL in CTRLs. In contrast, the modified SR‐X instruments A, B and C quantified CSF IL17A in 84.4%, 96.9% and 96.9% of CSF samples, respectively, with mean AD concentrations of 2.92fg/mL, 5.95fg/mL and 7.25fg/mL, respectively, and mean CTRL concentrations of 3.60fg/mL, 4.75fg/mL and 9.02fg/mL, respectively. Furthermore, the modified SR‐X instruments A, B and C all quantified IL17A in 100% of serum samples, with mean serum AD concentrations of 0.145pg/mL, 0.285pg/mL, and 0.249pg/mL, respectively, and mean CTRL concentrations of 0.300pg/mL, 0.406pg/mL and 0.362pg/mL, respectively. There were no statistically significant intra‐instrument differences between AD compared with CTRLs. All three modified SR‐X serum concentrations showed a strong positive statistically significant correlation with HD‐X serum concentrations. All three modified SR‐X serum concentrations also showed a strong positive statistically significant correlation with one another. Finally, all three modified SR‐X instruments showed no correlation between serum and CSF concentrations, in line with previous literature.

**Conclusion:**

Increasing the bead reading efficiency of the SR‐X exhibits increased sensitivity compared with the standard Simoa HD‐X instrument, allowing the quantification of IL17A in CSF and a greater yield of serum samples.

